# Knee Injury and Osteoarthritis Outcome Score Patellofemoral Questionnaire: Psychometric Properties among Females of Kingdom of Saudi Arabia

**DOI:** 10.3390/ijerph19106058

**Published:** 2022-05-16

**Authors:** Msaad Alzhrani

**Affiliations:** Department of Physical Therapy and Health Rehabilitation, College of Applied Medical Sciences, Majmaah University, Al Majma’ah 11952, Saudi Arabia; m.alzhrani@mu.edu.sa

**Keywords:** KOOS-PF, knee, reliability, scale, test-retest

## Abstract

Patellofemoral joint osteoarthritis (PFJ-OA), being a subset of knee osteoarthritis (KOA), is evident in adults, and its prevalence is greater in women in Saudi Arabia too. To assess its disease dimensions, the ‘Knee Injury and Osteoarthritis Outcome Score Patellofemoral’ questionnaire (KOOS-PF) is frequently used to measure symptoms and function among the people with PFJ-OA. Cross-cultural validation is ongoing in several languages, and it needed to be validated among females in Arabic. Therefore, aiming to translate, cross-culturally adapt and validate its psychometric properties, a cross-sectional study was designed where the Ar-KOOS-PF-F was administered among 105 females. The demographic characteristics of recruited females were 51.62 (8.49) years and 30.12 (3.70) kg/m^2^. Cronbach’s alpha was used for internal consistency (IC) and the questionnaire was re-administered after 48 h to estimate the test–retest reliability (92 females, 87.61% compliance rate). Concurrent validity was also established with a visual analog scale (VAS). Factorial validity was established by principal component analysis (PCA). The psychometric properties were: excellent internal consistency of Cronbach’s alpha (α) = 0.930, intraclass correlation coefficient (ICC) for intra-ratter reliability = 0.960 (0.915–0.999), test–retest reliability, ICC = 0.893 (0.889–0.970), standard error of measurement (SEM) = 2.46, relative standard deviation/coefficient of variance (RSD/CV) = 29.9%, minimal detectable change (MDC%) = 22.96% and good concurrent validity with VAS (r = −0.783; *p* = 0.023). The best-fit four-factor model for confirming overall item communalities ranged from 0.529 to 0.867, which indicates moderate to high communalities, and confirms the homogeneity of Ar-KOOS-PF-F using PCA. The floor (0.9%) and ceiling effects (13.6%) were also within the limits. This scale can be used among females, as it has acceptable psychometric properties of scale validation.

## 1. Introduction

Knee osteoarthritis (KOA) is one of the chronic diseases worldwide impacting the general and joint mobility of patients [1,2], and women bear about 47% lifetime risk of developing KOA [3]. It is considered an active disease process, featured by joint destruction driven through both biomechanical and pro-inflammatory factors [4]. One recent review study stated that there is a relationship between systemic origin of osteoarthritis (OA) and the intestinal microbiota where the pro-inflammatory microbiome profile in OA patients may play a role in the severity of symptoms [5]. The cartilage changes were visualized via magnetic resonance imaging (MRI), and they were associated with elevated pain along with the subjective evaluation scores of the femoral-tibial symptoms [6]. Meanwhile, the diffuse pain in the patella (anteromedial areas) chiefly symbolizes the patellofemoral joint osteoarthritis (PFJ-OA) and is evident during running, squatting, stair activities [7,8,9]. The pain that is associated with periarticular muscle tissue in knee OA is due to the prevalence of myofascial trigger points (MTrPs), varying from 11% to 50% in different muscles of patients with mild to moderate painful knee osteoarthritis [10]. The PFJ-OA is evident in 20 to 30% of adult people aged 26 to 50 years having longstanding patellofemoral (PF) pain, and females are the primary victims of this [11,12]. One study concluded that the PFJ-OA is very common in Saudi patients [13] and more prevalent in Saudi Arabian females [14]. Valgus deformity will accelerate lateral PFJ-OA along with patellar dysplasia patella or tibial malrotation and direction or force of the quadriceps femoris [15]. Likewise, other health care providers, such as physiotherapists, also adapt various assessment tools to measure the degree to which impairments exists, the efficacy of therapeutic approaches and the prognosis. These tools were categorized into subjective and objective types based on the measurement properties [16].

‘Knee Injury and Osteoarthritis Outcome Score Patellofemoral’ (KOOS-PF) is one subjective type of questionnaire developed as an extension with the aim of estimating the immediate and long-term symptoms and function among people with PFJ-OA [17]. It is disease specific and measures the PFJ-originated knee discomforts. The KOOS-PF comprises eleven items, which extensively evaluate the PFJ-born symptoms, such as stiffness, pain, as well as quality of life (QOL) [17,18]. Its psychometric properties were well established along with acceptable limits of structural validity from the patients [19].

There are many questionnaires available in original English versions today to evaluate the knee symptoms [18]. The KOOS-PF is a tool to evaluate knee symptoms originating from PFJ-OA [19]. Moreover, the KOOS-PF concept of cross-cultural validation is ongoing in several languages across the globe, and this questionnaire has been validated in the Arabic language among male subjects [20]. To better serve the people of Kingdom of Saudi Arabia (KSA) diagnosed with the PFJ pain, which is more common in Saudi females [13,14], and help the patients better express their symptoms in their mother tongue [21] thus mandated the need to validate it in Arabic among females. Therefore, the objective of the study was to translate, cross-culturally adapt and validate the psychometric properties of KOOS-PF among females (Ar-KOOS-PF-F) of KSA.

## 2. Materials and Methods

### 2.1. Study Design

A cross-sectional study of observational design was conducted where the outcome and the exposure in the study participants were measured at one point in time based on the inclusion and exclusion criteria set for the study.

### 2.2. Study Setting

A total of 105 female patients with diagnosed chronic patellofemoral pain were recruited through the convenience sampling technique, and the data were collected at the department of Physical therapy, University hospital, Majmaah University, Al Majmaah city-11952, Riyadh province, Kingdom of Saudi Arabia (KSA), from 23 December 2021 to 13 February 2022. After a leap of 48 h, they were again asked to report it to ensure the reliability of KOOS-PF. Out of 105 female patients, only 92 (87.61% compliance rate) were reported for the second session. We considered a leap of 48 h (i.e., before starting analgesics) between two reporting trials to prevent bias due to analgesic effect and recall bias of KOOS-PF items. The numeric visual analog scale (VAS) was considered to evaluate the patellofemoral pain among the participants.

### 2.3. Participants

Patients with chronic patellofemoral pain were invited to participate in the study followed by an initial interview where the study was explained to the patients. The inclusion criteria were as follows: (1) those aged between 30 to 60 years, diagnosed by an orthopedic surgeon with patellofemoral pain at the outpatient department of the University hospital, referred to the physical therapy department on the same day, were considered. (2) Patients who were able to read and write in Arabic. Patients were excluded from the study who had a history of acute and chronic knee problems other than patellofemoral pain, and patients with morbid obesity with previous history of tibio-femoral OA presented with patellofemoral pain were also excluded from the study to avoid bias in the outcome measures. The Majmaah University for Research Ethics committee (MUREC) (HA-01-R-088) reviewed the referred application, and the ethical aspects were approved with the Ethics Number’ MUREC-Dec.22/COM-2021/16-2. The patients were asked for written informed consent before the start of the study protocol. After obtaining an informed consent, demographic details, such as age, and female patients’ body mass index (BMI) were estimated from their height and weight, and then the patients were asked to fill in the Ar-KOOS-PF-F questionnaire on the same day of the initial interview along with visual analog scale, which was used to measure their pain threshold.

### 2.4. Validation Protocol

The author has obtained the permission to use the instrument from the copyright holders [20]. KOOS-PF incorporates 1 question describing the symptoms and quality of life (QOL), whereas the remaining 9 describe the pain out of 11 items. Each question of KOOS-PF was provided with five options, ‘never to always’, while describing the disability. The specification of scoring direction is necessary for a better interpretation of results. Arab people usually offer prayers/Salat five times daily, and it primarily involves the knee joint and other joint activity. Thus, while validating the KOOS-PF, the prayer activities were taken into account alongside the Beaton guidelines [22].

The entire validation process was described in five stages. The first stage consisted of a process of translating the English KOOS-PF to Arabic language by T1 and T2 (informed and uninformed) translators. Language discrepancies were analyzed, and an Arabic draft (T-12) was synthesized in the second stage. During the third stage, the T-12 draft was re-translated into the English language by two English professors from KSA back to the first language (BT1 and BT2). The pre-final version of the draft was prepared in the fourth stage by considering the opinion and suggestions obtained from all four translators (T1, T2, BT1 and BT2) along with an orthopedic surgeon. Thus, the prepared pre-final draft was implemented on twenty natives of KSA diagnosed with patellofemoral pain and evaluated for language fluency and easy understanding of each item in relation to their knee issues. The opinions of all participants were taken into account by the panel and approved for inclusion of Arabic words ‘Sujud’ and ‘Rakaa’ based on necessity, as reported in previous research of KOOS-PF validation among males [20]. The word ‘Sujud’ is an act of bowing where the forehead touches toward the ground, and ‘rakha’ is kneel sitting during ‘Salat’.

In the fifth stage, once the participants had been satisfied with the translated KOOS-PF items, the final draft was prepared and pre-tested. Minor changes were incorporated and renamed as Ar-KOOS-PF-F.

### 2.5. Estimation of Psychometrics Properties

#### 2.5.1. The Cronbach’s α

To ensure the internal consistency (IC) of each item of Ar-KOOS-PF-F, the Cronbach’s α was considered to report the data. Good IC of the subscale was agreed between 0.7 and 0.9. Items scoring over 0.9 were considered excellent, whereas items scoring below 0.7 were discarded [23].

#### 2.5.2. Reliability

The difference among the trials (test and retest) was achieved with intra-class correlation coefficient (ICC) with 95% confidence interval (CI) [23,24]. For better test–retest reliability, the ICC value should be >0.80. During the intra-class correlation coefficient (ICC) analyses, a 48 h gap was maintained between the first and second trials in order to prevent therapeutic drugs from influencing the results.

#### 2.5.3. Correlation Matrix

Here, the scores of the domains of the scale are supposed to be between 0.3 and 0.8 matrix [25].

#### 2.5.4. Relative Standard Deviation/Coefficient of Variance (RSD/CV)

Two well-known techniques adapted for estimating the percentage of agreement were relative standard deviation/coefficient of variance (RSD/CV) [26] and minimal detectable change (MDC) [27]. This was estimated from standard deviation (SD) and mean, giving a ratio for variability. The two steps in the analysis of the MDC are measurement of the error (SEM), followed by the measurement.

#### 2.5.5. Validity

##### Construct Validity

The minimum sample size required to establish the construct validity of Ar-KOOS-PF-F with VAS was reported using the formulae [28], N = [(Zα + Zβ)/C]2 + 3 = 105; Where, C = 0.5 ∗ ln[(1 + r)/(1 − r)] = 0.3205; r = 0.31 (Expected construct validity); Zα = Z0.05 = 1.96 (1%-Type I error rate); Zβ = Z0.10 = 1.28 (10%-Type II error rate). Construct validity was extracted from baseline values (*n* = 105) by Spearman correlation test, which investigated the relation of Arabic-version Ar-KOOS-PF-F with numeric visual analog scale (VAS). If the correlation coefficients were above 0.70, they were considered strong; between 0.70 and 0.50, they were considered moderate; whereas below 0.50, they were considered weak [29].

##### Factorial Validity

Homogeneity of Ar-KOOS-PF-F was determined using principal component analysis (PCA). Sample size estimation was required as the first step in performing factor analysis and was based on the number of items included in the questionnaire. As recommended, 3 to 20 samples were required for each item [30,31,32], thus making the minimum of 33 to 220 samples to perform the factor analysis. Another recommendation reported that the minimum sample required for the factor analysis should be at least, *n* = 100 [33]. Hence, our recruited sample of *n* = 105 would satisfy both recommendations. Further, the sampling adequacy was verified using the Kaiser–Meyer–Olkin (KMO) measure > 0.5 for acceptable and 0.8 for meritorious [34,35] and the Bartlett’s test of sphericity (BTS) to confirm the fitness to run EFA for the items included. The factor solution was based on eigenvalue > 1 and scree plot in deciding the number of factors required to confirm factorial validity. Factor loading should yield a minimum of (≥0.32) as acceptable communalities, (≥0.5) as moderate communalities and (≥0.8) high communalities [36,37]. A 60% variance was considered as minimum acceptable to explain total item variance [38].

Each item of Ar-KOOS-PF-F was checked for its floor and ceiling effect. It was predefined that the statistically acceptable limits were derived from the highest or lowest possible score reported by 15% of the samples [39].

### 2.6. Statistical Analysis

The psychometric measurement properties of the translated version, such as internal consistency (Cronbach’s α), reliability, test–retest by intra-class correlation coefficient (ICC). Among the total subjects (*n* = 105), only 92 (87.61%) were reported and considered for retest reliability analysis, and 13 subjects were not reported (12.38%). Inter-item correlation matrix by Spearman’s correlation, coefficient of variance by standard deviation (SD) and mean followed by measurement of error (SEM). Construct validity was extracted from baseline values (*n* = 105) by Spearman’s correlation test. Factorial validity: homogeneity of Ar-KOOS-PF-F was determined using principal component analysis (PCA). The sampling adequacy was verified using the Kaiser–Meyer–Olkin (KMO) measure > 0.5. Bartlett’s test of sphericity (BTS) to confirm the fitness to run EFA for the items included was conducted using IBM-SPSS statistical (Version 24.0) software (IBM Corp., Armonk, NY, USA). The level of significance was set at *p* < 0.05.

## 3. Results

### 3.1. The Demographics and Baseline Data

The demographics and baseline data of 105 female subjects who were eligible and recruited for the validation of Ar-KOOS-PF-F, as shown in Table 1.

### 3.2. Internal Consistency

Internal consistency (IC) of Ar-KOOS-PF-F expressed in terms of Cronbach’s α = 0.93 for 11 questions of the Ar-KOOS-PF-F questionnaire. The resulting value of 0.93 lies within an acceptable range among each item, and the average is 0.96 with a lower and upper bound of 0.915 and 0.999, respectively. These values were found to become statistically significant once the female patellofemoral pain patients reported the Ar-KOOS-PF-F following a leap of 48 h since the first trial.

### 3.3. Test and Retest Reliability

The obtained ICC must be above 0.80 for a good reliability. Additionally, the 48 h leap is necessary to minimize the potential influence of pharmacotherapy, which might cause alteration in the retest results. The first session Ar-KOOS-PF-F scored 29.72 (9.91), while the retest scored 33.67 (11.24), along with the mean difference between the two scores of 3.95 (7.57), test–retest reliability of 0.893 with standard error of 2.46 (SEM) as shown in Table 2. Test–retest reliability by intra-class correlation coefficient (ICC) (*n* = 105) where 13 subjects did not report (12.38%) for retest due to their personal issues and hence were not considered for analysis, whereas, only 92 subjects completed the questionnaire after 48 h for retest, and the data were used for analyzing the test–retest reliability.

### 3.4. Inter-Item Correlation Matrix

The 0.3 × 0.8 matrix analysis was depicted in Table 3. The correlation analysis among each item of the Arabic-version KOOS-PF subscale was lower than 0.8 (Table 3). This study reports the lowest inter-item correlation among PF2 and PF1 items (0.316) and the highest among PF11 and PF9 items (0.740). None of the correlations exceeded the standard limits of 0.8, indicating a good degree of inter-item correlation.

### 3.5. Relative Standard Deviation/Coefficient of Variance (RSD/CV)

Based on the coefficient of variation (CV) of the Arabic-version KOOS-PF (22.96%) and the MDC% of 22.96%, Ar-KOOS-PF-F is within acceptable limits (<30%).

### 3.6. Construct Validity

VAS demonstrated a moderate degree of association with the Ar-KOOS-PF-F items (r = −0.783; *p* = 0.023).

### 3.7. Factorial Validity

**Figure 1:**
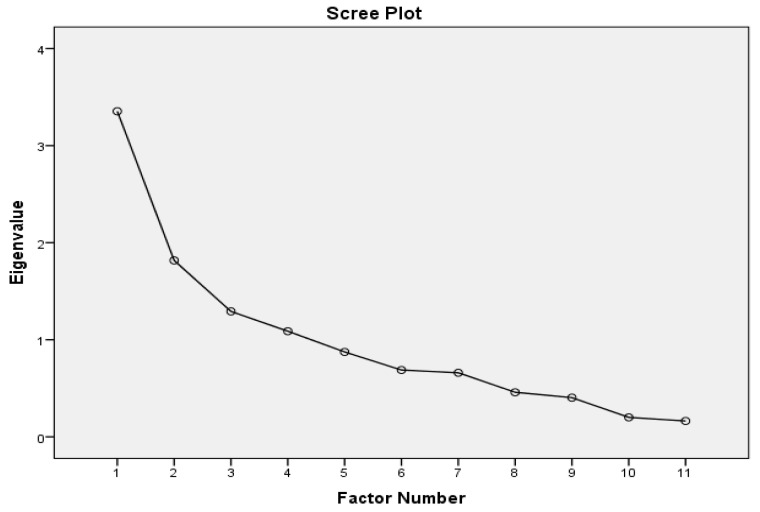
Scree plot confirming the factor number with eigenvalue (>1).

Sample adequacy (*n* = 105) confirmed by KMO measure was found to be 0.81, and it was considered meritorious [34,35], and BTS confirmed (Chi-Square = 386.162; *p* < 0.001) that the 11-item model was fit to run principal component analysis (PCA). Based on eigenvalue > 1 and scree plot (Figure 1), the four-factor model fits better to explain the data under the 11-item model with cumulative variance of 68.645% (Table 4), which was considered acceptable [38]. Factor loading for each item of the Arabic translated version of the 11-item Ar-KOOS-PF-F was tabulated in Table 5. It was evident that the minimum factor loading for the item was 0.352 and the highest was 0.811. As the factor-loading ranges between 0.352 and 0.811, it lies within the permissible limit to confirm factorial validity [33]. Overall, item communalities vary from 0.529 for the seventh item to 0.867 for the ninth item in Table 6, which yields moderate to high communalities [31,33]. Thus, the homogeneity of the Ar-KOOS-PF-F was determined using PCA.

### 3.8. Floor and Ceiling Effect

The minimum and maximum scores for every single item of Ar-KOOS-PF-F are pictured in Table 7. None of the items demonstrate the floor ceiling effect in our report. The values 0.9% and 13.9% were the lowest and highest floor effects opted for the PF6 and PF4 items. The values 3.8% and 13.6% were the lowest and highest ceiling effects opted for the PF1 and PF11 items, respectively, as shown in Table 7.

## 4. Discussion

The objective of this study was to translate the original version of KOOS-PF to Arabic, cross-culturally adapt and validate the psychometric properties of the Arabic version of Ar-KOOS-PF-F among females of the Kingdom of Saudi Arabia; having high ICC, test–retest reliability, along with the acceptable levels of good construct validity against VAS and acceptable factorial validity to confirm the homogeneity emphasizes the utilization of Ar-KOOS-PF-F with PFJ pain during activities of daily living involving knee joint functions. The internal consistency (0.93) reported in this study was similar to Ar-KOOS (0.87) and KOOS-PF for Arabic males (0.81) [20,39,40,41]. In this study, the individual domain pain (item No: PF2, PF3, PF4) exhibited an internal consistency of 0.93; ADL was 0.87; sports and recreation were 0.91 along with quality of life (QOL), which was 0.95. Thus, the obtained values for pain and QOL were nearer to the KOOS-Short Form (KOOS-SF), which holds an internal consistency of 0.75–0.95 and 0.9 for pain and QOL, respectively. Ar-KOOS-PF-F inter-item correlation as shown in (Table 3) was also mimicking the 12-item KOOS-SF (pain—0.43 and QOL—0.44) [42,43]. Thus, items of the Ar-KOOS-PF-F demonstrate a good internal consistency among them. The resultant Cronbach’s α of Ar-KOOS-PF-F measured in females is the same as the KOOS-PF original version (0.86) and falls within acceptable limits [19,39,40,42].

The creators of KOOS-Short Form were quoted on various short form versions of it having the diverged contents for the provision of domain-oriented intra-articular values [40,41], which assist in the conceptualization and validation of Ar-KOOS-PF-F. One previous study aimed at the KOOS Urdu validation study also supports this Arabic-version Ar-KOOS-PF-F, where the stiffness (PF1) (Cronbach’s α = 0.88) is marginally above the symptoms (Cronbach’s α = 0.930) [44]. Domains such as pain (0.89) and QOL (0.79) were supported in our results by maintaining the final scores within the standard psychometric boundaries, and a good level of internal consistency is always within the limits [29].

The Ar-KOOS-PF-F internal consistency (Cronbach’s α = 0.93) of our study was within the acceptable limits, which were in line with three other study reports, portraying a good internal consistency and correlation among the items. This was exactly the same as what was reported in the Spanish version of KOOS-PF [43] and higher when compared to previous KOOS-PF validation among the male population [20].

The average ICC (0.96) of Ar-KOOS-PF-F shows an excellent test–retest reliability and is above the original KOOS-PF (ICC = 0.86) [19] and Spanish KOOS-PF (ICC = 0.82) [45]. The ICC of Arabic-Version Long KOOS was 0.959 and higher for the symptoms (0.94), corresponding to PF1, pain (0.93) corresponding to PF2-10 and QOL (0.93) corresponding to PF11 of Arabic-version KOOS-PF [41].

Meanwhile, the Indian Urdu version of long-form-KOOS demonstrates that the ICC of symptoms (0.96) corresponds to PF-1 item and is below the ICC of pain (0.978), corresponding to PF2-10 items, and is approximate to the ICC of QOL (0.968), corresponding to PF11 item of the Arabic-version KOOS-PF [44].

In this study, Ar-KOOS-PF-F demonstrates a moderate degree of association with VAS (r = −0.783; *p* = 0.023), which is a good association when compared to our validation of males [20] and (r = 0.71) of the Spanish KOOS-PF reported with the Spanish Kujala score [45]. These findings were very similar to the correlation between Arabic-version KOOS-SF and VAS (pain—0.71, symptoms—0.59 and QOL—0.64) [41]. The current study result also demonstrates the 22.96% of MDC for Ar-KOOS-PF-F, which also lies below 30% [27] and is higher than the MDC of Arabic-version long-form KOOS [41].

All 11 questions in Ar-KOOS-PF-F failed to touch the floor or ceiling limits [18], which implies more than 15% of participants failed to choose the lower score (0) and the greatest score (4). Similar results were also evident in a study with original KOOS-PF [19]. Future research shall be executed on the responsiveness property in females.

### Strength and Limitations

Ar-KOOS-PF-F scale for measuring the PFJ pain syndrome among Muslim females of KSA was confirmed by factor analysis and was adapted with a special consideration to their prayer activities (Salat); this is the strength of the study. Additionally, the time period among the trials for estimating the test and retest for reliability was very minimal to nullify the analgesic effect of the pharmacotherapy for the same.

Due to the cultural barrier, male and females were not evaluated together within the hospitals of KSA. The KOOS-PF was validated among males by the previous author; hence, only females were recruited in this process of validation of measurement properties of Ar-KOOS-PF-F. Additionally, hence, the outcomes of Ar-KOOS-PF-F are applicable only to the female natives of KSA.

## 5. Conclusions

Ar-KOOS-PF-F is proved to be a valid, reliable tool for evaluating the female patients with patellofemoral pain in KSA, as the score results of its measurement properties are within the recommended acceptable limits.

## Figures and Tables

**Table 1:** Baseline characteristics of female patients with patellofemoral pain (*n* = 105).

Characteristics	Mean (SD) Range
Age: below 40 years	12 (11.42%)
Age: above 40 years	93 (88.57%)
Age (Years)	51.62 (8.49) (34–66)
BMI (Kg/m^2^)	30.12 (3.70) (21.5–36.4)
KOOS-PF	29.72 (9.91) (4.55–47.73)
VAS	6 (1.4) (4–9)

SD: Standard Deviation, BMI: Body Mass Index, KOOS-PF: The Knee Injury and Osteoarthritis Outcome Score Patellofemoral, VAS: Visual Analog Scale.

**Table 2:** Test and retest reliability and measurement error of Ar-KOOS-PF-F.

Subscale	Baseline Score Mean (SD)	Re-Test Score Mean (SD)	Mean Difference	ICC (95% CI)	SEM
KOOS-PF	29.72 (9.91)	33.67 (11.24)	3.95 (7.53)	0.893 (0.889–0.970)	2.46

ICC: Intra-Class Correlation Coefficient; SD: Standard Deviation; CI: Confidence Interval; SEM: Standard Error of Measurement.

**Table 3:** Inter-item correlation matrix for Ar-KOOS-PF-F.

Items	PF1	PF2	PF3	PF4	PF5	PF6	PF7	PF8	PF9	PF10	PF11
PF1	-										
PF2	0.316	-									
PF3	0.352	0.408	-								
PF4	0.443	0.491	0.433	-							
PF5	0.421	0.350	0.437	0.345	-						
PF6	0.324	0.402	0.544	0.492	0.401	-					
PF7	0.531	0.376	0.436	0.453	0.577	0.464	-				
PF8	0.429	0.437	0.424	0.442	0.511	0.421	0.488	-			
PF9	0.440	0.496	0.326	0.385	0.429	0.621	0.495	0.521	-		
PF10	0.325	0.357	0.419	0.329	0.485	0.457	0.393	0.365	0.305	-	
PF11	0.323	0.387	0.363	0.517	0.595	0.475	0.416	0.069	0.740	0.693	-

**Table 4:** Four-factor model of principal component analysis for 11 items.

Items	Eigenvalues		
	Total	% of Variance	Cumulative Percentage of Variance Explained
1	3.354	30.490	30.490
2	1.817	16.518	47.008
3	1.292	11.747	58.755
4	1.088	9.891	68.645
5	0.875	7.951	76.596
6	0.688	6.258	82.854
7	0.660	6.001	88.855
8	0.459	4.171	93.025
9	0.403	3.667	96.693
10	0.200	1.823	98.515
11	0.163	1.485	100.000

**Table 5:** Four-factor model with factor loading under 11 items.

Items	Four-Factor Model			
	1	2	3	4
PF_11_1	0.811			
PF_9_1	0.782		0.352	
PF_4_1	0.712		0.372	
PF_6_1	0.676			0.368
PF_7_1	0.533	0.484		
PF_3_1		0.691		
PF_10_1	0.464	0.675		
PF_1_1	0.366	0.668		
PF_2_1	0.447		0.688	
PF_8_1			0.423	0.751
PF_5_1	0.435		0.342	0.499
Extraction Method: Principal Component Analysis.				

**Table 6:** Factor loading of 11-item communalities.

Items	Factor Loadings
PF_1_1	0.656
PF_2_1	0.697
PF_3_1	0.625
PF_4_1	0.664
PF_5_1	0.592
PF_6_1	0.673
PF_7_1	0.529
PF_8_1	0.806
PF_9_1	0.867
PF_10_1	0.701
PF_11_1	0.740

**Table 7:** The mean, standard deviation, total correlation of items along with floor and ceiling effect of individual items in Ar-KOOS-PF-F (*n* = 105).

S No		Mean	Standard Deviation	Item–Total Correlation	Floor Effect N (%)	Ceiling Effect N (%)
1	KOOS-PF1	2.65	0.58	0.652	0 (0)	4 (3.8)
2	KOOS-PF2	2.62	0.77	0.733	0 (0)	9 (8.6)
3	KOOS-PF3	2.85	0.74	0.587	0 (0)	8 (7.3)
4	KOOS-PF4	2.93	0.81	0.814	13.9	13 (11.8)
5	KOOS-PF5	2.99	0.88	0.771	0 (0)	11 (10)
6	KOOS-PF6	2.78	1.01	0.750	1 (0.9)	14 (12.7)
7	KOOS-PF7	2.62	0.67	0.807	0 (0)	7 (6.4)
8	KOOS-PF8	2.65	0.89	0.800	0 (0)	14 (12.7)
9	KOOS-PF9	3.00	0.95	0.692	0 (0)	14 (12.7)
10	KOOS-PF10	2.55	0.73	0.705	0 (0)	9 (8.6)
11	KOOS-PF11	3.2	0.70	0.729	0 (0)	15 (13.6)

Ar-KOOS-PF-F: The Arabic Knee Injury and Osteoarthritis Outcome Score Patellofemoral in Females. Floor and ceiling effect values are in percentage (%).

## Data Availability

Data are available on request from the author. Data are not publicly available due to privacy and ethical concern.

## References

[1] Grässel S., Muschter D. (2020). Recent advances in the treatment of osteoarthritis. F1000Research.

[2] Heidari B. (2011). Knee osteoarthritis prevalence, risk factors, pathogenesis and features: Part I. Casp. J. Intern. Med..

[3] Neogi T. (2013). The epidemiology and impact of pain in osteoarthritis. Osteoarthr. Cartil..

[4] Guilak F., Fermor B., Keefe F.J., Kraus V.B., Olson S.A., Pisetsky D.S., Setton L.A., Weinberg J.B. (2004). The role of biomechanics and inflammation in cartilage injury and repair. Clin. Orthop. Relat. Res..

[5] Romero E.A.S., Oliva E.M., Pérez J.L.A., Pérez S.M., Turroni S., Marchese L., Villafañe J.H. (2021). Relationship between the Gut Microbiome and Osteoarthritis Pain: Review of the Literature. Nutrients.

[6] Hunter D.J., March L., Sambrook P.N. (2003). The association of cartilage volume with knee pain. Osteoarthr. Cartil..

[7] McConnell J. (1996). Management of patellofemoral problems. Man Ther..

[8] Powers C.M. (1998). Rehabilitation of patellofemoral joint disorders: A critical review. J. Orthop. Sports Phys. Ther..

[9] Halabchi F., Abolhasani M., Mirshahi M., Alizadeh Z. (2017). Patellofemoral pain in athletes: Clinical perspectives. J. Sports Med..

[10] Romero E.A.S., Carnero J.F., Villafañe J.H., Calvo-Lobo C., Sáez V.O., Caballero V.B., Val S.L., Pedersini P., Martín D.P. (2020). Prevalence of Myofascial Trigger Points in Patients with Mild to Moderate Painful Knee Osteoarthritis: A Secondary Analysis. J. Clin. Med..

[11] Collins N.J., Oei E., de Kanter J.L., Vicenzino B., Crossley K.M. (2019). Prevalence of Radiographic and Magnetic Resonance Imaging Features of Patellofemoral Osteoarthritis in Young and Middle-Aged Adults with Persistent Patellofemoral Pain. Arthritis Care Res..

[12] Hinman R.S., Lentzos J., Vicenzino B., Crossley K.M. (2014). Is Patellofemoral Osteoarthritis Common in Middle-Aged People with Chronic Patellofemoral Pain?. Arthritis Care Res..

[13] Al-Arfaj A., Al-Boukai A.A. (2002). Prevalence of radiographic knee osteoarthritis in Saudi Arabia. Clin. Rheumatol..

[14] Mohammad W.S., Elsais W.M. (2021). The Epidemiology of Patellofemoral Pain in Majmaah, Saudi Arabia. Asian J. Pharm. Res. Health Care.

[15] Alejandro A.L., Yenima G.L., Guadalupe L.L., Mercedes L.L. (2013). Patellofemoral osteoarthritis. Rev. Cuba. Ortop. Traumatol..

[16] Ateef M., Kulandaivelan S., Tahseen S. (2016). Test-retest reliability and correlates of 6-minute walk test in patients with primary osteoarthritis of knees. Indian J. Rheumatol..

[17] Roos E.M., Lohmander L.S. (2003). The Knee injury and Osteoarthritis Outcome Score (KOOS): From joint injury to osteoarthritis The Knee injury and Osteoarthritis Outcome Score (KOOS). Health Qual. Life Outcomes.

[18] Rodriguez-Merchan E.C. (2012). Knee instruments and rating scales designed to measure outcomes. J. Orthop. Traumatol..

[19] Crossley K.M., Macri E.M., Cowan S.M., Collins N.J., Roos E.M. (2018). The patellofemoral pain and osteoarthritis subscale of the KOOS (KOOS-PF): Development and validation using the COSMIN checklist. Br. J. Sports Med..

[20] Ateef M. (2020). Measurement properties of the knee injury and osteoarthritis outcome score patello-femoral questionnaire in saudi arabians. PeerJ.

[21] Lindquist K.A., MacCormack J.K., Shablack H. (2015). The role of language in emotion: Predictions from psychological constructionism. Front. Psychol..

[22] Beaton D.E., Bombardier C., Guillemin F., Ferraz M.B. (2000). Guidelines for the process of cross-cultural adaptation of self-report measures. Spine.

[23] Rattray J., Jones M.C. (2007). Essential elements of questionnaire design and development. J. Clin. Nurs..

[24] Juul T., Søgaard K., Davis A.M., Roos E.M. (2016). Psychometric properties of the Neck OutcOme Score, Neck Disability Index, and Short Form-36 were evaluated in patients with neck pain. J. Clin. Epidemiol..

[25] Juul T., Søgaard K., Roos E.M., Davis A.M. (2015). Development of a patient-reported outcome: The neck outcome score (noos)-content and construct validity. J. Rehabil. Med..

[26] Simonsen J.C. (1995). Coefficient of variation as a measure of subject effort. Arch. Phys. Med. Rehabil..

[27] Lee P., Liu C.-H., Fan C.-W., Lu C.-P., Lu W.S., Hsieh C.L. (2013). The test-retest reliability and the minimal detectable change of the Purdue pegboard test in schizophrenia. J. Formos. Med. Assoc..

[28] Hulley S., Cummings S., Browner W., Grady D., Newman T. (2013). Designing Clinical Research: An Epidemiologic Approach.

[29] Terwee C.B., Bot S.D., de Boer M.R., van der Windt D.A., Knol D.L., Dekker J., Bouter L.M., de Vet H.C. (2006). Quality criteria were proposed for measurement properties of health status questionnaires. J. Clin. Epidemiol..

[30] Everitt B.S. (1975). Multivariate analysis: The need for data, and other problems. Br. J. Psychiatry.

[31] Cattell R.B. (1978). The Scientific Use of Factor Analysis in Behavioral and Life Sciences.

[32] Comrey A.L., Lee H.B. (1992). A First Course in Factor Analysis.

[33] Mvududu N.H., Sink C.A. (2013). Factor Analysis in Counseling Research and Practice. Couns. Outcome Res. Eval..

[34] Kaiser H.F. (1982). Educational and Psychological Measurement. Ser. Rev..

[35] Kaiser H.F., Rice J. (1974). Little Jiffy, Mark Iv. Educational and Psychological Measurement. Scientif. Res..

[36] Jackson P.R., Wall T.D., Martin R., Davids K. (1993). New measures of job control, cognitive demand, and production responsibility. J. Appl. Psychol..

[37] Swank J.M., Lambie G.W., Witta E.L. (2012). An exploratory investigation of the Counseling Competencies Scale: A measure of counseling skills, dispositions, and behaviors. Couns. Educ. Superv..

[38] Yong A.G., Pearce S. (2013). A Beginner’s Guide to Factor Analysis: Focusing on Exploratory Factor Analysis. Tutorial. Quantitat. Method. Psychol..

[39] Roos E.M., Roos H.P., Ekdahl C., Lohmander L.S. (1998). Knee injury and Osteoarthritis Outcome Score (KOOS)—Validation of a Swedish version. Scand. J. Med. Sci. Sports.

[40] Duncan R., Peat G., Thomas E., Wood L., Hay E., Croft P. (2009). Does isolated patellofemoral osteoarthritis matter?. Osteoarthr. Cartil..

[41] Alfadhel S.A., Vennu V., Alnahdi A.H., Omar M.T., Alasmari S.H., AlJafri Z., Bindawas S.M. (2018). Cross-cultural adaptation and validation of the Saudi Arabic version of the Knee Injury and Osteoarthritis Outcome Score (KOOS). Rheumatol. Int..

[42] Gandek B., Roos E.M., Franklin P.D., Ware J.E. (2019). A 12-item short form of the Knee injury and Osteoarthritis Outcome Score (KOOS-12): Tests of reliability, validity and responsiveness. Osteoarthr. Cartil..

[43] Gandek B., Roos E.M., Franklin P.D., Ware J.E. (2019). Item selection for 12-item short forms of the Knee injury and Osteoarthritis Outcome Score (KOOS-12) and Hip disability and Osteoarthritis Outcome Score (HOOS-12). Osteoarthr. Cartil..

[44] Ateef M., Kulandaivelan S., Alqahtani M. (2017). Cross-Cultural Validation of Urdu Version KOOS in Indian Population with Primary Knee Osteoarthritis. Int. J. Rheumatol..

[45] Martinez-Cano J.P., Vernaza-Obando D., Chica J., Castro A.M. (2021). Cross-cultural translation and validation of the Spanish version of the patellofemoral pain and osteoarthritis subscale of the KOOS (KOOS-PF). BMC. Res. Notes.

